# The Role of Urban Environment Design on Health During the COVID-19 Pandemic: A Scoping Review

**DOI:** 10.3389/fpubh.2022.791656

**Published:** 2022-04-29

**Authors:** Sara Faedda, Alessandro Plaisant, Valentina Talu, Giulia Tola

**Affiliations:** Department of Architecture, Design and Planning (DADU), University of Sassari, Sassari, Italy

**Keywords:** urban environment, physical health, mental health, COVID-19, urban design, scoping review, vulnerable inhabitants

## Abstract

The COVID-19 pandemic has had a significant impact on the ways and times of living and using urban spaces, specifically referring to the dimension of daily life. The restrictive measures introduced during the lockdown periods have necessarily led to a re-evaluation of proximity scale bringing particularly attention to issues relating to public transport and mobility and to the quality and distribution of open public spaces. This scoping review explores the relationship between the urban environment design and health referring to the constraints imposed by the COVID-19 pandemic in the period from 2020 to 2021, with two main objectives: (i) to investigate the recurring urban design topics and issues related to the spatial and social needs stressed by the emergency; (ii) to identify the urban design measures both experienced during the health emergency and proposed in view of a post-COVID urban and territorial planning as they are considered impactful on health promotion. The search strategy was based on a set of keywords searched in two electronic databases which allowed the identification of a total of 1,135 contributions. After defining the eligibility criteria, we proceeded to the screening process concluded with the inclusion of 19 studies. The analysis of the contributions led to the systematization of six main urban topics—and to the corresponding spatial requirements and project proposals—highlighted as relevant and supportive in terms of the promotion of inhabitant's public health: (i) transport, mobility and accessibility; (ii) green and outdoor spaces; (iii) public and pedestrians' spaces; (iv) care services and health network; (v) communications; (vi) public and business services. The resulting framework is useful for guiding healthy city planning toward public policies, tools, regulations, urban measures, and emergency contrast provisions, that contribute to increasing the effectiveness in terms of safety and well-being.

## Introduction

The research addresses the correlation between urban design and health with particular reference to the constraints deriving from the COVID-19 pandemic.

The purpose is two-fold, that is to deepen the urban planning aspects that have affected both the spatial and social realm during the emergency period, and to identify a framework of relevant urban projects and interventions in the promotion of urban health.

The COVID-19 pandemic has had a significant impact on the ways and times of living and using urban spaces, specifically referring to the dimension of daily life.

The restrictive measures introduced during the lockdown periods have necessarily led to a re-evaluation of proximity scale, as the well-known and discussed model of “15-Minutes City” (1), bringing particularly attention to issues relating to public transport and mobility and to the quality and distribution of open public spaces (2).

In fact, the possibility of accessing essential services and resources has become a crucial requirement of our settlements with respect to mobility limitations imposed by COVID-19, resulting in a worsening of social and territorial disparities together with a need to re-act and reprogram planning processes starting from the local scale.

A significative example in this regard is represented by the recent statistics collected by the United Nations according to which only about half of the world population resides in areas served by public transport services within a pedestrian radius of 500 meters (3) and the urban global area devoted to street and outdoor public spaces is of 16% against the desirable 30% ((3), p.48, 49).

The interrelation between the characteristics of urban settlements and Public Health have been largely addressed in the last decades consolidating the awareness about the held part of built environment planning and policies design on wellbeing (4).

In the recent scenario of total or semi-lockdown, remote working, scarce off-line social interaction, limitations on the accessibility of services and public spaces, spatial attributes should be analyzed with reference to the effects of the outdoor built environment associated to mental health benefits (5–7).

Pandemic spatial and social dynamics have further emphasized these aspects by renewing the relevance of adopting a healthy city oriented approach (8, 9) together with operative programs that guide policies and urban projects toward the highest achievable overall state of health, such as the “Healthy Cities Network” (10, 11), even to pursue the global Sustainable Development Goals with particular reference to the 11th of fostering “inclusive, safe, resilient and sustainable cities” (12).

Starting from the previous considerations, the research question of the present scoping review addresses the relationship between the urban environment design and health referring to the constraints imposed by the COVID-19 pandemic between the years 2020 and 2021 with two main objectives described below.

The first is to investigate the recurring urban design topics and issues related to the spatial and social needs stressed by the emergency.

The second is to identify and to describe urban planning and design aspects both experienced during the health emergency by COVID-19 and proposed in view of a post-COVID urban and territorial planning as they are considered effective and impactful on health promotion—understood as the achievement of a state of well-being that contemplates both the physical, mental and social realms (13).

The topic addressed in this contribution is to be considered relevant mainly in consideration of the fact that non-pharmaceutical interventions—including the reconfiguration and reorganization of urban spaces—are and will remain essential even in the presence of an adequate vaccination coverage for reducing virus transmission (14).

## Methods

The review was carried out consistently with the methodological process proposed by the “Joanna Briggs Institute Manual for Evidence Synthesis” with specific reference to the scoping reviews' framework outlined by Arksey and O'Malley (15) and the recent updates reported by Peters et al. (16).

The scoping review was developed according to the five-stages methodological procedure of Arksey and O'Malley (15) whose steps are the following:

(i) definition of the research question
(ii) identification of relevant studies
(iii) studies selection
(iv) charting the data
(v) collating, summarizing and reporting the results

They are described in detail below.

### Definition of the Research Question

The first stage proposed by Arksey and O'Malley (15) operative framework refers to the definition of the research question. This is a fundamental step in defining a structured methodology oriented toward an equally defined objective and, as also underlined by Arskey and O'Malley [(15), p.23], to clarify which factors reported by the studies are to be considered of greatest interest for the research (e.g., the population, the methods of intervention, the outcomes, etc.).

Therefore, the scoping review here presented aims to investigate the relationship between the urban environment design and health referring to the constraints imposed by the COVID-19 pandemic, to identify and describe the city's design aspects that had a significant impact in terms of health promotion.

### Identification of Relevant Studies: Search Strategy

The review adopted a search strategy based on a set of keywords consistent to the main topic investigated and the research question (Table 1).

**Table 1:** Search settings.

**Search topics**	**Search terms**
Built environment	Built environment, urban environment, urban setting, urban features, environmental characteristic, housing, settlement, neighbourhood
Public health	COVID, health

The selected search terms were combined using Boolean operators thus obtaining the search string summarized below (see Supplementary Data Sheet 1).

Built Environment OR Urban Environment OR Urban Setting OR Urban Features OR Environmental Characteristic OR Housing OR Settlement OR Neighborhood AND Health AND COVID.

In this context, a clarification is necessary. The choice to adopt only the term “COVID” in the development of the string, instead of using also other related thematic keywords (such as “SARS-CoV-2,” “2019-nCoV,” etc.) derives from the need to refine the scoping review from the very first steps of the research, selecting from the huge amount of contributions only those relevant in the field of urban planning.

The studies searching was carried out through two electronic databases, Scopus and PubMed which led to the identification of a total of 1,135 of contributes.

### Studies Selection: Inclusion and Exclusion Criteria

The third stage was the definition of the eligibility criteria to proceed with the screening process. Then inclusion and exclusion criteria have been defined as outlined in Table 2.

**Table 2:** Eligibility criteria.

**Inclusion criteria**	**Exclusion criteria**
The studies are peer-reviewed articles, and grey literature like reports and design guidelines	Reviews, editorials, comments, conference proceedings and dissertations
The studies refer to the general population without any limitation in age and gender	Thematic inconsistency with the objective of the research question
The studies outline design features, spatial requirements, projects and policies at the urban scale for promoting health in the context of the restrictions imposed by the COVID-19 pandemic	No abstract availability together with inconsistency with the research topic according to title checking Theoretical studies with mainly analytical approach and purposes

Peer-reviewed studies and relevant gray literature consistent with the eligibility criteria were included. Other literature reviews and non-original studies (editorials, comments, conference proceedings and degree theses) were excluded, as well as contributions resulting out of topic with respect to the research question. Among these there are both studies related to other disciplines (such as, for example, studies in the medical and epidemiological fields) and papers investigating the broad topic of housing sometimes focusing attention on questions like access to housing, homelessness, mental health related issues to the confinement or specific architectural aspects involved in well-being promotion not overall consistent with the urban scale addressed by the research question. Furthermore, the studies with no abstract and contextually inconsistent with the research question according to title checking were also excluded.

Included studies outlined design features, spatial requirements, projects, and policies recognized as relevant to the promotion of health in the context of the restrictions imposed by the COVID-19 pandemic. Finally, the selected articles concern the general population, without any limitation in age and gender.

**Figure 1:**
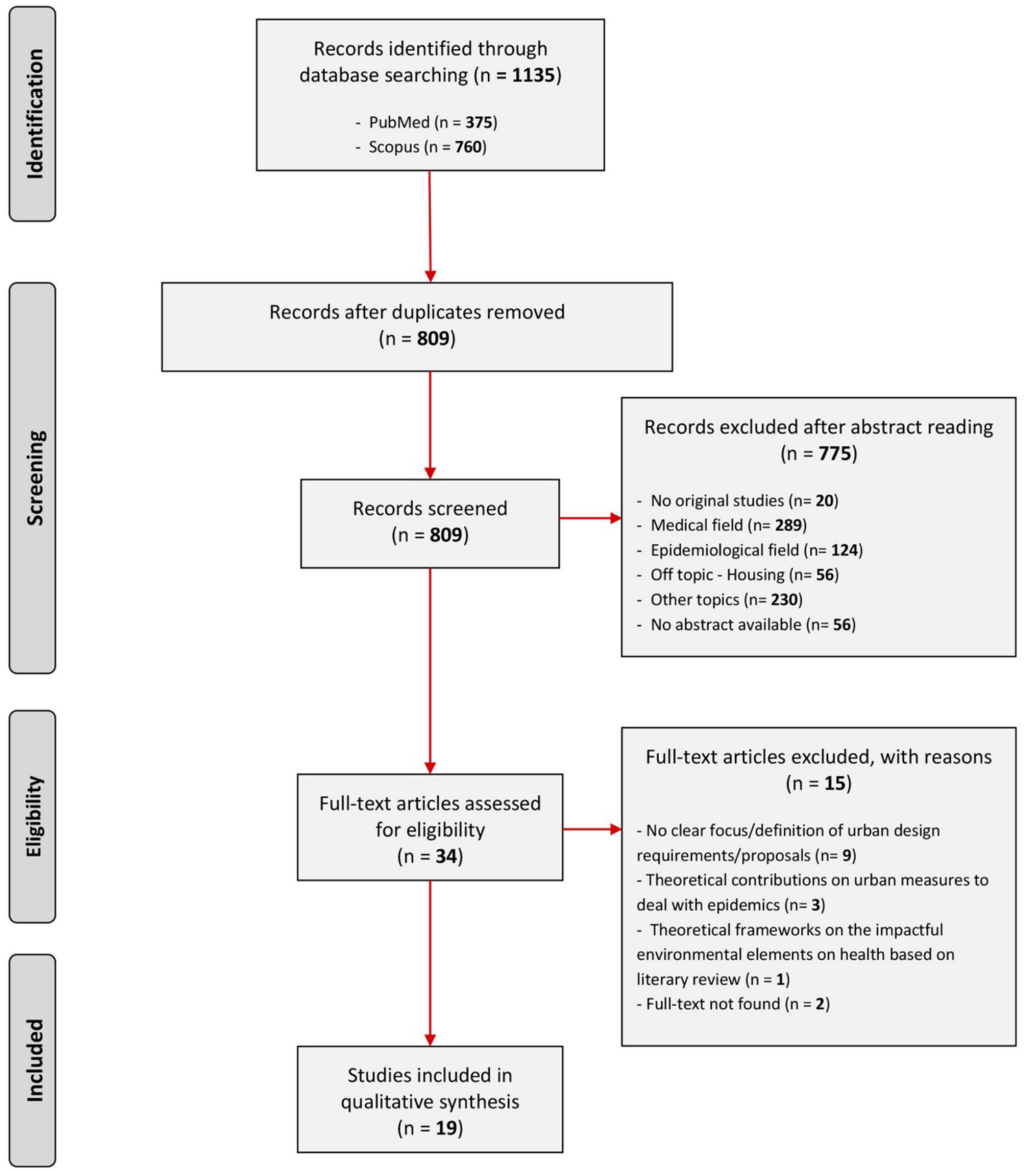
The studies selection process is reported according to the Preferred Reporting Items for Systematic Reviews and Meta-Analyses (PRISMA) flowchart model (17). Studies selection: inclusion and exclusion criteria.

The screening process was carried out by two reviewers according to a double pass and reported according to the Preferred Reporting Items for Systematic Reviews and Meta-Analyses (PRISMA) flow diagram (17) (Figure 1).

The first was based on the check through title and abstract to obtain an initial and consistent selection of articles by excluding those that were not relevant in the first place.

The second checked the articles by reading through the full texts previously selected.

All the contributions focusing exclusively on the topic of the design of home environments and without any reference to the urban dimension were excluded, in order to bring out the studies which outline design solutions in the area of urban planning and design, during and after the pandemic.

Furthermore, all the contributions with analytical purposes were excluded, to include therefore only studies with a design and operational approach.

After the exclusion of 326 duplicates, 809 articles were screened through title and abstract checking, 34 full-text studies were assessed for eligibility and a total of 19 were finally included as they met the inclusion criteria.

In fact, the full-text analysis led to the exclusion of a second group of studies, namely 15, as their outcomes didn't fully satisfy the research question addressed. Specifically, *n* = 9 studies did not report a clear definition of urban design requirements; *n* = 3 provided a theoretical contribution on general urban measures to deal with epidemics; *n* = 1 developed theoretical and broad frameworks on the impactful environmental elements on health based on literary review; finally, *n* = 2 the full-texts were not found.

### Charting the Data

The full texts screening stage ended with the systematization of the data through the development by the authors of a data extraction grid based on the Joanna Briggs Institute data charting model (16, 18).

The main and proper information extracted are summarized (see Supplementary Table 1) whose last column reports the urban policies and projects topics pointed out as relevant in relationship to the new spatial needs emphasized by the COVID pandemic. Supplementary Table 1 reports, respectively, the following data: the main studies features (author/s, publication year, study country and study design); the participants information (age range, gender, number of participants); the data collection method/s; the design scale the studies referred to (urban, territorial, neighborhood, and proximity scale); the study outcomes and finally the policies and projects topics or requirements.

### Collating, Summarizing, and Reporting the Results

The last step involved systematizing the outcomes of the 19 included studies.

The contents reported in the last column of Supplementary Table 1 have been organized through a categorization into the main and recurring topics of policies and projects further articulated in the specific interventions identified and suggested by the studies' authors (Table 3). Specifically, this part is deepened in the Discussion section.

**Table 3:** Urban spatial requirements and project proposals to promote Public Health at the time of COVID-19.

**Design topic**	**Requirements**	**Policies and projects/actions**	**References**
Transport Mobility Accessibility	- Increase the safety and the effectiveness of the public transport system and the overall mobility network - Improve active transport by focusing on cycle and pedestrian mobility - Promote accessibility and safety enhancing the reachability to urban spaces - Increase the accessibility of cultural sites - Promote “flexibility of city schedules” - Reduce noise level - Promote a proximity dimension in the form of 20 min neighbourhoods	- Limit vehicular traffic on residential streets - Promote shared streets projects by closing neighbourhood street to non-local traffic - Introduce speed limits - Introduce share mobility policies and programs - Support the use and adoption of alternative transport modes such as electric cars, e-scooters - Full street closure to all public motor traffic - Tactical and temporary interventions at the micro-scale to design and test new road lanes devoted to cycling and to general active mobility	(19–28)
Green and outdoor spaces	- Improve exposure to green spaces - Encourage and promote the access and use of existing residential outdoor spaces and nearby greenery	- Provide neighbourhood green areas - Encourage and promote outdoor activities and play - Implement the green infrastructure in urban areas together with agricultural opportunities - Increase the adoption of nature-based solutions	(19, 24, 29–35)
Public and pedestrians'spaces	- Reorganize streets, squares, parks according to a proximity scale - Promote a multi-functional design for outdoor spaces ensuring safety distance - Improve cities walkability	- Extend sidewalks, even by removing parking, to make walking/exercise safer - Convert parking into public space for people - Provide temporary patios for seating/ensure physical distancing - Organize a system of vacant lots in a network of creative public spaces - Design of little “circle islands” acting as micro public spaces by using materials like spray chalk, temporary paint or vinyl graphics which helps to keep the right safety distancing	(19, 21, 22, 36)
Care services and health network	- Improve accessibility to primary healthcare services	- Design and plan public transport routes and/or schedules according to the demand of healthcare services - Improve the design of services for the elderly - Integrate the existing environmental emergency plans, with those prepared for the health emergencies	(20, 27)
Communication	- Improve stakeholders' awareness and information	- Use visual supports to provide the necessary information to ensure public hygiene - Promote the development of smart cities with a digitization process of urban information	(20, 36)
Public and business services	- Promote spatial solutions to allow the commercial and restorative activities to meet the needs of neighbourhood's inhabitants by guaranteeing the observance of the safety measures - Strengthen the neighbourhood social infrastructure	- Acquisition of additional external space for commercial activities by subtracting parking lots and extending pedestrian space where possible by placing level platforms that also allow for additional space to be obtained to ensure physical spacing - Transform vacant lot in dining garden for communities - Foster the availability of portable kiosk network that can be rented from the business activities or communities for a designated period - Define a neighbourhood services' plan according to population density and neighbourhood size - Provide a diverse mix of uses locally (shops, education facilities, sports, and social activities)	(20, 24, 36, 37)

## Result

The studies included were 19 out of 1,135 initially obtained and they are all published in the time span between 2020 and 2021.

Furthermore, as expected, the use of research keys relating to COVID and health associated with the environment subject matter have intercepted numerous studies concerning other issues correlated to the pandemic. Some of the most recurrent concern the controversial aspects about the spread of COVID and their contributing factors—both environmental and socio-spatial—together with the topic of the social inequalities and the global poverty deepened by COVID-19, the public health system management at different levels.

A substantial part of the studies has been conducted in Europe, specifically in Italy (*n* = 3), Bulgaria (*n* = 1), Greece (*n* = 1) and Sweden (*n* = 1). A second group of 5 has been carried out in UK while a third including 2 contributions in the USA. Finally, the last six studies are, respectively, from Australia (*n* = 1), Canada (*n* = 2), China (*n* = *2*), and Turkey (*n* = 1).

### Study Design and Data Administration

Included studies have been carried out according to many different methodological frameworks. In summary, the study design and related approaches found are the following: case studies and literature based research (19–24, 29); secondary based research (25, 30); cross sectional studies (26, 31–33); mixed-methods approach based on literature review combined with data collection and analysis (27, 28) surveys (34, 35, 37) and co-design processes (37).

The prevailing method for data collection and management was through the administration of online questionnaires (26, 32–35, 37) as the studies were mostly conducted during the period of major restrictions.

The remaining studies in which this information could be extracted used questionnaires managed with telephone interviews (31), adopted crowdsourcing data collection methods (28) and gathered panels of experts (36).

The prevailing studies population consisted of general population, especially young students and adults aged 18-50 and, in some cases, of children and teenagers around 5-17 years old.

### Built Environment Design Scale and Settlement Type

More than half of the studies referred to the broad scale of urban environment mapping out policies, projects, and design requirements to improve public health as a whole (19–22, 24, 25, 27–29, 31–33, 35, 36).

A group of four studies explored the neighborhood dimension focusing on the element and characteristic which play a role on the promotion of physical and mental health of inhabitants (26, 30, 34, 37).

Finally, one study addressed the subject of the research by looking at the type of settlement, particularly the informal one (23).

### Studies Outcomes

A total of 11 out of 19 studies made explicit and well-defined urban design features, spatial requirements, guidelines, and policies that have assumed or will have a significant role in terms of promoting urban public health in a pandemic context (19–21, 23–26, 29, 31, 33, 37).

In some cases, however, we need to revise or summarized the contents without impair the original meaning (22, 27, 30, 34–36).

Lastly, in the remaining two studies it was necessary to deduct them as they were not explicit (28, 32).

## Discussion

The included studies provide urban design features, spatial requirements, urban projects or policies that in the specific context outlined by the pandemic have assumed an important and supportive role in the promotion of inhabitant's public health.

Precisely, some studies report policies and projects experienced in the first phases of the COVID-19 emergency or suggested interventions to address the consequents spatial and social needs of our cities in the short and long-term (19–25, 27, 29, 36).

On the other hand, some studies were based on data collection to investigate urban strategies and elements which produced improvement effects on health during the epidemic (26, 28, 30–35, 37).

Starting from the last column of Supplementary Table 1, we have identified the relevant and recurring themes, each of which was then deepened in the corresponding spatial requirements, related actions and project proposals and reported in Table 3.

The main issues addressed by the studies regard, respectively: (i) transport, mobility and accessibility; (ii) green and outdoor spaces; (iii) public and pedestrians' spaces; (iv) care services and health network; (v) communications; (vi) public and business services. These are described in detail below.

*Transport, mobility, and accessibility* (19–28).

The first theme focuses on the need to improve the public transport network and the cycling and pedestrian infrastructures, to promote a greater level of safety given the spatial needs due to COVID-19 emergency and to provide more opportunities to foster active mobility. The main projects and actions suggested concern, respectively:

- the improvement of public space devoted to pedestrian by encouraging the adoption of shared streets policies;
- the introduction of speed limits, especially in residential areas;
- the design and development of short-term tactical interventions to provide wider available surface to active mobility.

*Green and outdoor spaces* (19, 24, 29–35).

The second topic reported by about half of the studies concerns the city's green and outdoor spaces and the important role that the exposure to them play on health well-being. The actions suggested can be summarized as follow:

- the need to guarantee wide access to green areas by providing neighborhoods green infrastructures to encourage outdoor activities;
- the enhance of adopting nature-based solutions in urban areas.

*Public and pedestrians' spaces* (19, 21, 22, 36).

The third item addressed the design of proximity public spaces as a whole and their walkability level focusing on the necessity to rethink their organization in order to ensure the safety distances and prioritize pedestrian uses. The main proposals concern:

- the extension of sidewalks surfaces by acting on the reduction of car park;
- the set-up of temporary micro-public spaces according to tactical urbanism approach (38) by taking advantage of vacant lots to give the possibility of seating and spending time outdoor by ensuring physical distancing.

*Care services and health network* (20, 27).

The next theme regards the public health and the need to guarantee and improve the access to its services, considering in particular:

- the necessity to strengthen the availability of services devoted to the most vulnerable groups of inhabitants, particularly the elderly;
- the implementation of a greater accordance between the time planning of public transport routes with the schedules and demand of healthcare services;
- the update and integration of existing environmental emergency plans with those planned for health emergencies.

*Public and business services* (36).

The second-last topic covers the design of the commercial and restorative activities' nearby spaces to address the needs and requests of neighborhood inhabitants by guaranteeing the observance of the safety measures. The main action proposal can be summarized as follow:

- here too, the gaining of additional outdoor space by widening the pedestrian space in parking lots, renewing vacant lots where possible and installing platforms and portable kiosk are suggested.

*Communications* (20, 36).

Finally, the last aspect recognizes a great importance to the acquisition process of awareness and information of stakeholders through a clear, accessible, and effective communication. Thus, the proposed actions are listed below:

- the adoption of visual support and signage aimed at providing all the significant information to comply with the basic public hygiene rules to deal with the COVID-19 pandemic;
- the start of a process of information digitization functional to reach wider different groups of inhabitants.

The current work presents some limitations which should be pointed out.

The first concerns the choice of databases which has been limited to two of the different ones available, so this may have led to the loss of some studies.

Secondly, some issues regarding the methodology adopted in the process of selecting contributions must be specified: (i) on the one hand, the choice to include only papers which, among the different dimensions of the built environment, only deal with the urban scale; (ii) on the other hand, the sole inclusion of operational studies with the exclusion of contributions with analytical purposes and of other types of material such as conference proceedings, editorials and other reviews. These methodological aspects may have equally affected the outcomes of the present scoping review as they involve a certain degree of subjectivity.

Thirdly, it should be borne in mind that the topic dealt with here is extremely topical and therefore constantly updated, especially if we consider the publication of studies and research subsequent to those analyzed by us whose publication took place by May 2021.

## Conclusion

In conclusion, the aim of this scoping review was to provide a cognitive overview on the topic concerning the relationship between urban planning and public health promotion at the time of COVID-19.

Most of the studies emphasize the need to (re)consider the neighborhood scale by encouraging urban measures and policies able to promote a proximity spatial and social dimension in order to guarantee crucial public health requirements further brought to attention by the pandemic, such as the reachability and accessibility to public spaces and relevant services and functions of everyday life.

Closely related to what has been said above, another important aspect that emerged refers to the possibility and need of promoting micro-transformations, therefore acting according to a tactical approach (38) to trigger lasting processes able to address the new spatial and social needs stressed by the pandemic.

The scoping review has systematized the knowledge and evidence available on a topic currently even more debated and subject to continuous updates. Therefore, the research is to be considered an open contribution that can be implemented both starting from the inclusion of further areas not explored here, such as that of the architectural scale and indoor environment, and from the integration of more recent studies that can contribute to the updating and deepening of the work here presented.

## Data Availability Statement

The datasets presented in this study can be found in online repositories. The names of the repository/repositories and accession number(s) can be found in the article/Supplementary Material.

## Author Contributions

AP, GT, and VT: conceptualization. SF and GT: data curation and investigation. GT and VT: methodology and writing—review and editing. GT: writing—original draft preparation. All authors have read and agreed to the published version of the manuscript.

## Funding

The APC was funded by: a) the 2019 University Research Grant of the University of Sassari Fondo di Ateneo per la Ricerca 2019 dell'Università degli Studi di Sassari, and b) the research project RUES - Rigenerazione Urbana ed Energie Sociali (Urban Regeneration and Social Energies), funded by Fondazione di Sardegna (budget of the year 2016 for research projects with peer review, based on the financial resources provided by Fondazione di Sardegna) and co-financed with Legge Regionale 7 /2007 of Sardinia Region (Italy) for the year 2016.

## Conflict of Interest

The authors declare that the research was conducted in the absence of any commercial or financial relationships that could be construed as a potential conflict of interest.

## Publisher's Note

All claims expressed in this article are solely those of the authors and do not necessarily represent those of their affiliated organizations, or those of the publisher, the editors and the reviewers. Any product that may be evaluated in this article, or claim that may be made by its manufacturer, is not guaranteed or endorsed by the publisher.

## Supplementary Material

Supplementary Table 1 Data of included studies.

Supplementary Data Sheet 1 Search string.
