# Supplementary material for: The Role of Urban Environment Design on Health During the COVID-19 Pandemic: A Scoping Review
Source: Front Public Health. 2022 Apr 29;10:791656. doi: 10.3389/fpubh.2022.791656 (PMC9099229; doi:10.3389/fpubh.2022.791656)
Supplement: Supplementary Table 1 — Data of included studies. [file Table_1.pdf]

**Author(S) (Year)**	**Study Country**	**Study Design**	**Participants** Age Range, Gender	**Administration**	**Built Environment Design scale/ Settlement Type**	**Studies Outcome**	**Policies and Projects Topics/Requirements**
**Ahsan, M. M. (2020)**	Turkey	Secondary based Research	N.A.	N.A.	Urban environment	Decisions and measures taken by government in urban environment perspective	▪ Provide accommodation to homeless people<br>▪ Ensure safer transportation systems<br>▪ Quarantine neighbourhood or building
**Barbarossa, L. (2020)**	Italy	Overview of case studies	N.A.	N.A.	Urban environment	Local government policies and strategies concerning post-COVID sustainable mobility	Practices based on tactical urbanism principles:<br>▪ Enhance non-motorized mobility removing motor traffic from residential streets<br>▪ Provide public spaces and services<br>▪ Extending pavements to make walking/exercise safe<br>▪ Create safe access routes on foot and bike<br>▪ Create safe public spaces and green areas at the neighbourhood scale<br>▪ Policies and interventions to improve cycling and pedestrian mobility<br>▪ Provide new cycling lane<br>▪ Provide share mobility programs<br>▪ Public spaces renewal<br>▪ Reorganize streets, squares, parks at a human scale
**Capolongo, S., Rebecchi, A., Buffoli, M., Appolloni, L., Signorelli, C., Fara, G. M., D'Alessandro, D. (2020)**	Italy	Literature based research	N.A.	N.A.	Urban environment	Possible solutions in the short and medium-long term to respond to problematic issues related to the pandemic	***Immediate Actions***<br>▪ Program the flexibility of city schedules<br>▪ Plan a smart and sustainable mobility network<br>▪ Define a neighbourhood services' plan according to population density and neighbourhood size<br>▪ Develop a digitization of the urban context, promoting the smart communities for monitoring and information purposes<br>▪ Re-think the accessibility to the places of culture and tourism<br><br>***Medium-long period actions:***<br>▪ Re-think building typologies, fostering the presence of semi-private or collective spaces<br>▪ Renovate the basic care services' network<br>▪ Integrate the existing environmental emergency plans, with those prepared for the health emergencies.<br>▪ Improve stakeholders' awareness
**Chen, Y., Jones, C., Dunse, N. (2021)**	China	Cross sectional study	*n*=937 participants; age 18 - 34 years old	Online questionnaire	Neighbourhood scale	Identification of neighborhood impactful elements to inhabitants' mental health	▪ Perception of neighbourhood noise level

**DeLange Martinez, P., Nakayama, C., Young, H. M. (2020)**	Baltimore, USA	Co-design approach	Stakeholder committee composed by experts in various fields including public health, urban planning and design, transportation	Constitution of a panel of experts for validating the design concepts	Urban environment	An "Ideas Guidebook" for the identification of ten design concepts to meet the spatial needs arising from Covid-19 emergency	▪ "Curblet Commons": convert parking into public spaces for people<br>▪ Organize the public space of the street according to a "circle islands system" thus designing micro-public spaces on the pavement to limit the space according to safety distancing<br>▪ Enhance an "hygiene system" to reduce disease transmission by using visual and spatial representation<br>▪ "Make ApART": organize a system of vacant lots in which people can be creative (art classes, performance)<br>▪ Organize the street space at the human scale<br>▪ "The food court": transform vacant lot in dining garden for communities<br>▪ "inFront of House": propose a framework of spatial elements right outside a business place to be used like an extension of the sidewalk or "portable kiosk" that can be rent by businesses or communities
**Dzhambov, A. M., Lercher, P., Browning, M. H. E. M., Stoyanov, D., Petrova, N., Novakov, S, Dimitrova, D. D. (2020)**	Bulgary	Survey	Students (*n*=323)	Online questionnaire	Neighbourhood scale	Requirements to promote physical and mental health	▪ Ensure green exposure
**Fischer, J., Winters, M. (2021)**	Canada	Case studies research	N.A.	N.A.	Urban environment	Interventions to re-design streets and integration with existing active transportation infrastructure	▪ Sidewalk expansion by removing parking<br>▪ Ensure safe access to parks/services<br>▪ Local streets closed to non-local traffic (shared streets)<br>▪ Full street closure to all public motor traffic<br>▪ Promotion of active transportation<br>▪ Reallocation of new bike line<br>▪ Temporary patios for seating/ensure physical distancing
**Guida, C., Carpentieri, G. (2021)**	Milan, Italy	Mixed-method approach:<br>1: Systematic literature review<br>2: GIS-based procedure<br>3. Results visualization	N.A.	N.A.	Urban environment	Policies to improve accessibility to primary healthcare services for elderly people	▪ Design public transport routes and/or schedules according to the demand of healthcare services<br>▪ Improve road and public transport network<br>▪ Improve services designed for the elderly and their needs<br>▪ Re-allocate medical resources within the existing structures<br>▪ Improve accessibility to primary healthcare services
**Gunn, L. D. (2020)**	Australia	Literature based research	N.A.	N.A.	Urban environment	Policies and interventions in order to promote sustainable urban mobility and improve cities liveability	▪ Promote healthy and sustainable forms of transport<br>▪ Improve cities walkability by widening sidewalks<br>▪ Decrease roads speed limits<br>▪ Close streets to allow safe accessibility to pedestrian and cyclist<br>▪ Provide safe cycling infrastructure<br>▪ Encourage the use of emerging transport modes such as electric cars, e-scooters

**Hong, J., McArthur, D., Raturi, V. (2020)**	Glasgow, UK	Mixed methods approach:<br>1. Literature review<br>2. Explanation of data and Analytical Model (case study)<br>3. Empirical result from regression model	N.S.	Crowdsourced cycling data collected from the activity-tracking app Strava	Urban environment	Changing in cycling intensity during lockdown according to the different types of cycling infrastructure	▪ Provide safe cycling infrastructure<br>▪ Increase in cycling<br>▪ Improve road safety to promote cycling<br>▪ Guarantee accessibility to parks by cycling<br>▪ Provide temporary cycling lanes on roads to encourage people to cycle more
**Hubbard, G., Den Daas, C., Johnston, M., Murchie, P., Thompson, C. W., Dixon, D. (2021)**	Scotland	1. Cross-sectional study<br>2. Studies review	Study 1: 2969 participants (median age 54 years);<br>Study 2: 502 participants (median age 53 years);	4-item Questionnaire conducted by telephone interviews	Territorial and urban scale	Impact of accessibility to outside spaces on people mental health	▪ Improve access to public green space<br>▪ Improve access to residential outside space<br>▪ Guarantee good quality housing<br>▪ Offer private access to shared residential space by operating a timed rotation for different households<br>▪ Encourage the use of existing residential outside space and public green space
**Kamalipour, H., Peimani, N. (2020)**	UK	Literature review	N.A.	N.A.	Informal settlements	Policies and projects to respond the implications of Covid-19 pandemic on informal settlements	Tactical and temporary interventions at the micro-scale
**Lõhmus, M., Stenfors, C. U. D., Lind, T., Lauber, A., Georgelis, A. (2021)**	Sweden	Cross-sectional study	Adults inhabitants<br>(n=2060; age ≥20 years)	Online survey	Urban environment	Impact of greenness exposure on people habits and mental health during Covid-19 pandemic	▪ Ensure access to nearby greenery<br>▪ Increase the use of nature based solutions to improve public health
**Miao, J., Zeng, D., Shi, Z. (2021)**	Wuhan, China	Mixed method approach:<br>1.Survey<br>2.Direct observation	Adults inhabitants<br>(*n*=3031, from 943 neighbourhoods)	Online questionnaire	Neighbourhood scale	The role of neighbourhood social infrastructure and social cohesion on residents mental health	▪ Increase public spending on neighbourhood organizations<br>▪ Strengthen the neighbourhood social infrastructure Provide opportunities for volunteering and other forms of social participation
**Mitra, R.<br>Moore, S. A.<br>Gillespie, M.<br>Faulkner, G.<br>Vanderloo, L. M.<br>Chulak-Bozzer, T.<br>Rhodes, R. E.<br>Brussoni, M.<br>Tremblay, M. S.<br>(2020)**	Canada	1. Literature review<br>2. Secondary based research	Children and youth<br>(*n*=1472; age 5-17 years)	N.S.	Neighbourhood scale	Requirements to promote active behaviour at the neighbourhood scale	▪ Ensure access to opportunities for outdoor activity, play and recreation<br>▪ Prioritize outdoor play space to improve children movement

**Poortinga, W., Bird, N., Hallingberg, B., Phillips, R., Williams, D. (2021)**	UK	Survey	UK citizens (*n*=5566)	Online questionnaire and qualitative interviews	Public and private green spaces	The role of public and private green space during the Covid-19 pandemic	▪ Guarantee access to public green space and private garden
**Pozoukidou, G. Chatziyiannaki, Z. (2021)**	Greece	Case studies research	N.A.	N.A.	Urban environment	Policies and actions implemented by three different cities based on proximity concept	▪ Protect biodiversity and natural assets<br>▪ Promote social mobility to enhance social cohesion<br>▪ Ensure neighbourhoods and suburbs inclusion<br>▪ Build effective governance, strong leadership and collaborative partnerships<br>▪ Promote local living in the form of 20 min neighbourhoods<br>▪ Promote the development of a diverse mix of uses locally (shops, education facilities, sports and social activities)<br>▪ Promote active mobility like walking or cycling in combination with public transport<br>▪ Promote the conversion and safety of roads to more accessible and pedestrian friendly infrastructure<br>▪ Ensure accessibility of public and green spaces
**Rice, L. (2020)**	UK	Literature based research	N.A.	N.A.	Urban environment	Definition of urban design policies and project to promote public health	▪ Expand the presence and function of nature in urban areas<br>▪ Provide urban agricultural opportunities<br>▪ Implement green infrastructure
**Tomasso, L. P., Yin, J., Laurent, J. G. C., Chen, J. T., Catalano, P. J., Spengler, J. D. (2021)**	USA	Cross-sectional study	*n*=529<br>[female (75%), white non-Hispanic (82%), and of slightly younger age (age 25–34 = 29%) although no age category was below 10.6%)]	Online questionnaire	Urban environment	Measures and projects to improve green exposure to individuals during the period of restrictions	▪ Re-introduce nature into urban areas to improve contact with nature<br>▪ Demand management and distributed access to public nature areas<br>▪ Promote green exercise as a safe, preventative health behaviour<br>▪ Invest in neighbourhood pedestrian green infrastructure

NA: Not Applicable
NS: Not Specified
