# Supplementary material for: The Role of Urban Environment Design on Health During the COVID-19 Pandemic: A Scoping Review
Source: Front Public Health. 2022 Apr 29;10:791656. doi: 10.3389/fpubh.2022.791656 (PMC9099229; doi:10.3389/fpubh.2022.791656)
Supplement: Supplementary Data Sheet 1 — Search string. [file Data_Sheet_1.pdf]

Supplementary Material A

“Search string”

Search topics: built environment; public health.

Search terms: Built environment, urban environment, urban setting, urban features, environmental characteristic, housing, settlement, neighbourhood, covid, health.

PubMed search string:
built environment"[Title/Abstract] OR "urban environment"[Title/Abstract] OR "urban setting*"[Title/Abstract] OR "urban features"[Title/Abstract] OR "environmental characteristic"[Title/Abstract] OR "housing"[Title/Abstract] OR "settlement*"[Title/Abstract] OR "neighborhood"[Title/Abstract]) AND "health"[Title/Abstract] AND "covid"[Title/Abstract].
